# Fetal Electrocardiogram Signal Extraction Based on Fast Independent Component Analysis and Singular Value Decomposition

**DOI:** 10.3390/s22103705

**Published:** 2022-05-12

**Authors:** Jingyu Hao, Yuyao Yang, Zhuhuang Zhou, Shuicai Wu

**Affiliations:** Department of Biomedical Engineering, Faculty of Environment and Life, Beijing University of Technology, Beijing 100124, China; haojy@emails.bjut.edu.cn (J.H.); yoga3y@emails.bjut.edu.cn (Y.Y.); zhouzh@bjut.edu.cn (Z.Z.)

**Keywords:** fetal ECG signal extraction, FastICA algorithm, singular value decomposition, wavelet mode maximum method, QRS waves

## Abstract

Fetal electrocardiograms (FECGs) provide important clinical information for early diagnosis and intervention. However, FECG signals are extremely weak and are greatly influenced by noises. FECG signal extraction and detection are still challenging. In this work, we combined the fast independent component analysis (FastICA) algorithm with singular value decomposition (SVD) to extract FECG signals. The improved wavelet mode maximum method was applied to detect QRS waves and ST segments of FECG signals. We used the abdominal and direct fetal ECG database (ADFECGDB) and the Cardiology Challenge Database (PhysioNet2013) to verify the proposed algorithm. The signal-to-noise ratio of the best channel signal reached 45.028 dB and the issue of missing waveforms was addressed. The sensitivity, positive predictive value and F1 score of fetal QRS wave detection were 96.90%, 98.23%, and 95.24%, respectively. The proposed algorithm may be used as a new method for FECG signal extraction and detection.

## 1. Introduction

Fetal electrocardiograms (FECGs) record the changes of cardiac action potential in the conduction process, offering reliable beat-by-beat information of fetal heart rates, and providing clinicians with fetal health information such as intrauterine hypoxia [1]. FECG signals are more sensitive than fetal heart monitor based on ultrasound Doppler with respect to fetal heart monitoring, and can better reflect conditions such as fetal intrauterine hypoxia [2]. However, FECG signals often see interference by maternal ECG (MECG) signals, which have much stronger amplitudes [3]. Therefore, the extraction of clean FECG signals is important and challenging.

Currently, FECG signals can be obtained by the invasive scalp electrode method and non-invasive abdominal electrode methods. The former can directly measure clean FECG signals, but only at the time of delivery. Non-invasive abdominal electrode methods collect mixed signals by placing electrodes on the pregnant women’s abdomen, which can be used for long-time monitoring during pregnancy. However, abdominal mixed signals of pregnant women are complex, comprising MECG signals, FECG signals, baseline drift, power frequency interference, and noises [4]. Blind source separation (BSS) [5] can be used to extract FECG signals from the abdominal mixed signals, but there is a large amount of residual noises in the extracted FECG signals.

Several FECG signal extraction methods have been explored, including empirical mode decomposition (EMD) methods [6], least mean square (LMS) error algorithms [7,8], singular value decomposition (SVD) methods [9], wavelet transform (WT) methods [10], independent component analysis (ICA) methods, and in particular fast fixed-point ICA (FastICA) algorithms [11]. Sarafan et al. [12] proposed a method combining ICA, template subtraction (TS), and extended Kalman filter (EKF) algorithms. They used three different ICA algorithms to extract MECG signals and FECG signals with residual noises. They then used EKF to filter out residual MECG signal components. Experimental results showed that TS combined with the FastICA algorithm yielded the best performance, with a high signal-to-noise ratio (SNR). The FastICA algorithm uses approximate negative entropy and Newton iterative methods to reduce the amount of computation, which has the advantage of fast convergence and is a widely used signal separation method [5]. However, its convergence performance is greatly affected by the initial weight [13]. Therefore, Yuan et al. [14] improved the conventional FastICA algorithm by introducing an overrelaxation factor to process the initial weight in the Newton iterative algorithm, which reduced the average iteration times from 55 to 15. However, there were residual noises in the extracted FECG signals and some waveforms were missing. The SVD algorithm can effectively separate each component of mixed signals. It constructs vector matrices using abdominal mixed ECG signals, and then obtains ECG signal estimation corresponding to each singular value. In this work, we proposed a new method combining FastICA and SVD algorithms for FECG signal extraction. Experimental results showed that the proposed algorithm had good performance in SNR and addressed the issue of missing waveforms.

**Figure 1:**
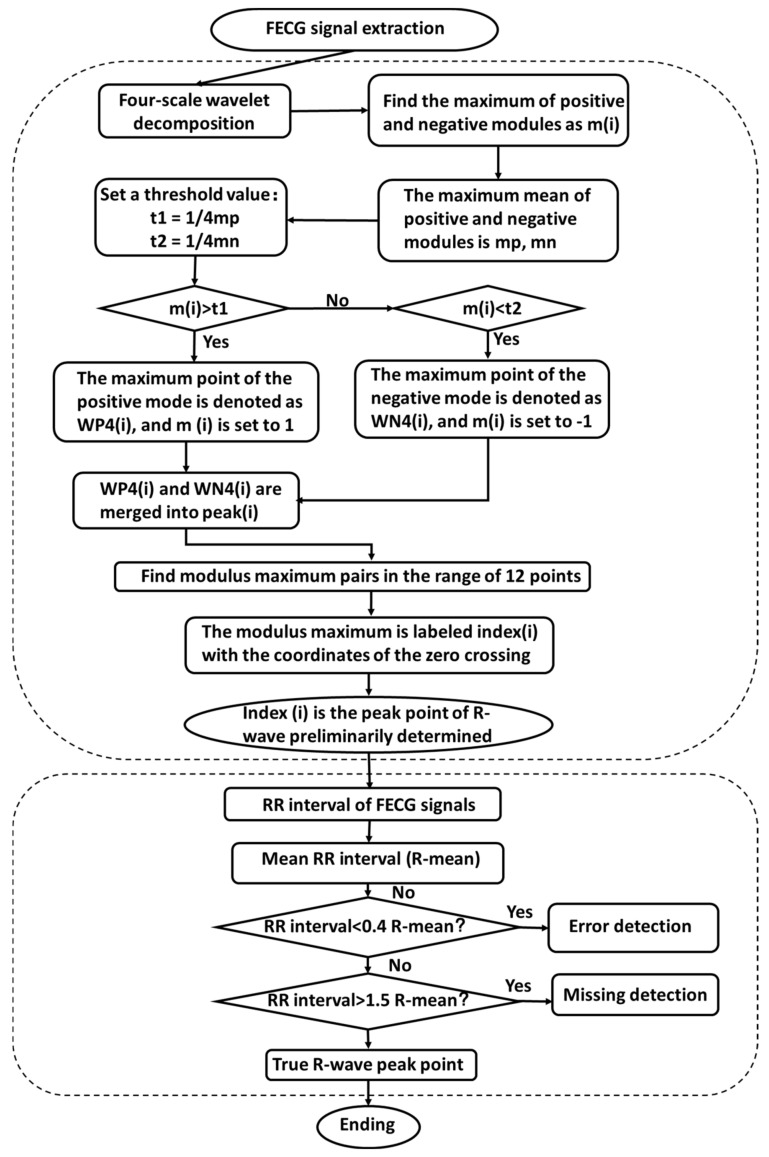
R-wave detection of ECG signals.

For QRS wave detection of extracted FECG signals, current methods mainly include neural network methods [15,16,17,18], template matching algorithms [19], and wavelet mode maximum methods [20]. Neural network methods have a high accuracy, but they take a long time to train the model. The template matching method requires a variety of probability distributions and is sensitive to high frequency noises. Wavelet mode maximum methods are simple in implementation and have a wide range of applications. Therefore, we employed the improved wavelet mode maximum method to detect the key features of extracted FECG signals (Figure 1), including QRS waves and ST segments. Finally, we integrated these extraction and detection algorithms into a user-friendly graphical user interface (GUI) to provide systematic support for the diagnosis of fetal health.

## 2. Materials and Methods

### 2.1. Datasets

The datasets used in this study were from two publicly available databases. The first database is the abdominal and direct fetal ECG database (ADFECGDB, https://physionet.org/physiobank/database/ADFECGDB, accessed on 6 May 2022). They were obtained by the Department of Obstetrics of the Silesia Medical University through the KOMPOREL system. The data were collected from five pregnant women of 38–41 weeks of gestation and had five pieces of data recordings (R01, R04, R07, R08, and R10). Each recording contained four signals from the maternal abdomen and one signal directly from the fetal head. The sampling frequency was 1000 Hz, and the sampling time was 5 min. The second database is the clinical ECG dataset for the PhysioNet/Computing in Cardiology Challenge Database (PhysioNet2013, https://physionet.org/physiobank/database/challenge/2013/, accessed on 6 May 2022) [21]. The Challenge 2013 Training Set A (Challenge/2013/seta) consisted of 25 recordings. Each recording contained four mixed signals from the maternal abdomen, with a sampling frequency of 1000 Hz and collection time of 60 s.

### 2.2. FECG Signal Extraction Based on the FastICA and SVD Algorithm

Clinically, when one uses the abdominal electrode method to collect mixed ECG signals, there are often many artifacts or noises due to maternal and fetal movement or collector displacement [22]. Therefore, we used the data preprocessing method proposed by Dessì et al. [23] to suppress baseline drift, power–frequency interference, and pulse artifacts.

The FastICA algorithm is a fast optimization iterative algorithm, including kurtosis based, maximum likelihood based, and maximum negative entropy based methods. In this study, we used the FastICA algorithm based on maximum negative entropy. After de-mean and whitening of the preprocessed signals, we took the maximum negative entropy as a search direction and extracted the independent source signals. The FastICA algorithm has a high precision, but it has a disadvantage in separation speed. Its initial weight will affect the efficiency of iteration, making the resulting independent components slightly different. Therefore, we incorporated the overrelaxation factor into the Newton iterative algorithm to process the randomly generated initial weights, so that the average number of iterations was reduced [14]. However, the experimental results showed that there were some missing waveforms and residual noises. Therefore, we further improved our previous method [14], and the specific steps are as follows.

Step 1: Firstly, we used the SVD algorithm to decompose maternal abdominal mixed ECG signals and used the wavelet mode maximum method to detect R-peak of FECG signals (Section 2.3.1). According to the R-R interval, we constructed the reconstruction matrix *A* of FECG signals through equal period interpolation.

Step 2: We performed SVD operation on matrix *A* and reserved the largest singular values to obtain the signal estimation matrix *A’*. Then, we removed the data corresponding to the interpolated positions in matrix *A’* to obtain approximate estimates of FECG signals. However, the signals have residual noises and missing waveforms at the overlapping part of maternal–fetal signals.

Step 3: We used the FastICA algorithm to estimate MECG signals and noises from mixed signals as reference, and separated the noises from the FECG signals obtained by Step 2. By using this method, the residual noises of the FECG signals were reduced, and the issue of missing waveforms was addressed.

Step 4: Finally, we calculated the heart rate, R-R interval, and other parameters of MECG and FECG signals.

The singular value difference spectrum theory [24] shows that when two neighboring singular values have a large difference, the difference spectrum will have a peak value. It is indicated that the singular value has a mutation at the current position, and the maximum mutation point is the boundary point between the target signal and noises. In Step 2, we set the period of the one-dimensional MECG signals as n, from which m cycles were extracted to construct an m×n signal matrix *A*. Then, there were two orthogonal matrices: $U=\left[U_{1},U_{2},\ldots ,U_{n}\right]$ and $V=\left[V_{1},V_{2},\ldots ,V_{n}\right]$, so that

(1) $$A=U\sum V^{T}$$

where $\sum =\left[\mathrm{diag}\left(\delta_{1},\delta_{2},\ldots ,\delta_{p}\right),0\right]$; $p=\min\left\{m,n\right\},\delta_{1}\geq \delta_{2}\geq \ldots \geq \delta_{p}\geq 0$ is called the singular value of *A*. Different singular values correspond to the energy concentration of different signal components in the signal, and the importance is positively correlated with the singular value. Each signal matrix is represented as the sum of a series of small matrices with rank 1, while the singular value measures the importance of the small matrices. A large singular value corresponds to the signal component with a high energy. When the signal energy is greater than the noise energy, the large singular value is retained and the remaining small singular value is zeroed. We used the ADFECGDB and PhysioNet2013 databases to evaluate the number of optimal singular values. When the number of singular values was set to 2, the FECG signals extraction performance was the best.

### 2.3. FECG Signal Detection Based on the Wavelet Mode Maximum Method

In order to detect and classify FECG signals, we need to analyze the key characteristic information for clinical diagnosis. Clinically, physicians generally assess the fetal health through the following instantaneous points and intervals:

- R-R interval: When R-R interval is too long and fetal heart is too slow, we can consider late hypoxia; when the R-R interval is unequal, e.g., the change of fetal heart rate is more than 25–30 bpm, we can preliminarily diagnose premature beats, cardiac arrest, irregular rhythm and other abnormalities, and we should do the next examination immediately.
- QRS waves: The normal duration of fetal QRS ranges from 0.02 s to 0.05 s. If it exceeds 0.05 s, it is abnormal. According to the duration and amplitude of QRS waves, fetal weights can be estimated, such as macrosomia or fetal growth retardation. For the fetus with hemolytic anemia, the severity of anemia can be determined [25].
- ST segment: ST segment is the potential line from the end of QRS waves to the beginning of T-waves, and the normal ST segment is equipotent. Metabolic acidosis should be considered when the ST segment is significantly depressed or elevated. Changes in ST segments are also an important indicator to evaluate whether the fetus is healthy [26]. Abnormal ST segments and T-waves of FECG signals indicate fetal electrolyte disorder, myocardial hypoxia, and abnormal myocardial metabolism.

#### 2.3.1. Wavelet Mode Maximum Method

According to different frequency distributions of ECG signals, the wavelet mode maxima method can decompose ECG signals at multiple scales. Each scale represents a different component of the ECG signals, and we identify the different waveform locations by determining the location of the over-zero point between pairs of modal maxima at different scales [10,27]. The principle of the wavelet mode maximum method is as follows. We set the wavelet function $\varphi \left(t\right)$ to be equivalent to the first derivative of the smooth function $\theta \left(t\right)$:

(2) $$\varphi \left(t\right)=\frac{d\theta \left(t\right)}{dt}$$

where $\varphi \left(t\right)$ satisfies $\int \varphi \left(t\right)dt=1$ and is a higher-order infinitesimal of $\frac{1}{1+t^{2}}$. The transform of $\theta_{s}\left(t\right)=a\theta \left(t/s\right)$ wavelet function is

(3) $$W_{x}\left(s,t\right)=x\left(t\right)*\varphi \left(t\right)=x\left(t\right)*\left(s\frac{d\theta_{s}\left(t\right)}{dt}\right)=s\frac{d\left(x\left(t\right)*\theta_{s}\left(t\right)\right)}{dt}$$

Equation (3) shows that the derivative of $W_{x}\left(s,t\right)$ is positively correlated with the derivative of $x\left(t\right)$ smoothed by $\theta \left(t\right)$. On the scale s, the modulus maximum after wavelet transform corresponds to the inflection point of $x\theta_{s}\left(t\right)$. There is a correlation between the modulus maximum and the mutation point.

#### 2.3.2. QRS Wave Detection

We first detected the peak of R-waves, and then detected the starting point and ending point of QRS waves. The steps of R-wave detection are as follows.

Step 1: Firstly, we chose the appropriate wavelet function. A quadratic spline wavelet was used to approximate the signal by using a set of conic curves. The spline wavelet is symmetric, tightly supported, and has linear phase, and the signal processed by it will not be distorted. Therefore, we used orthogonal quadratic spline wavelets to decompose FECG signals into four scale wavelets. The wavelet transformed signals will generate different mode maximum pairs at different scales, and the zero crossings of the mode maximum pairs will correspond to R-waves. By comparing the information on the four scales, it was found that the R-wave energy was mainly distributed at scales 3 and 4, and the fourth scale was stronger. Therefore, the fourth scale was used as the main signal of R-wave detection.

Step 2: We took all positive and negative modulus maxima of the fourth scale, calculated the mean values, and set appropriate thresholds according to the mean values. The positive mode maxima greater than the threshold were reserved, and the negative mode maxima less than the threshold were reserved, and the thresholds were set to 1 and −1, respectively. The positive mode maxima and negative mode maxima satisfying the conditions were denoted as *WP*4(*i*) (*i =* 1, 2, *…*) and *WN*4(*i*) (*i =* 1, 2, *…*), respectively. They were combined as *Peak*(*i*) (*i =* 1, 2, *…*). Finally, we found the points on scale 4 with modulus maximal value pairs *Peak*(*n*) *=* −1 and *Peak*(*n + k*) *=* 1.

Step 3: The peaks and valleys of FECG signals correspond to a pair of positive and negative mode maximum pairs on the scale. If there was an unmatched point, it would be wrongly detected and should be deleted. The FECG signal periods are generally 0.02 s to 0.05 s, so it could be found among 12 sampling sites. The R-wave peaks detection process is shown in Figure 1.

Step 4: False detection and missed waveform detection. Before searching, we cycled through the whole interval to filter out some useless elements. We found the maximum difference between adjacent elements, *MD*, and if there existed a known *M* and *M ≥ MD*, we filtered it out and adjusted the search interval.

Step 5: We calculated the mean R-R interval of the signal, *Ṝ*; If the R-R interval was less than 0.4 × *Ṝ*, the detection was a false detection. The improved dichotomy method was used to adjust the threshold, and steps 2 to 4 were repeated. On the contrary, if the R-R interval was greater than 1.5 × *Ṝ*, the detection was a missed detection. The improved dichotomy method was used to adjust the threshold, and steps 2 to 4 were the same.

Q-waves and S-waves are high-frequency and low-amplitude waveforms in ECG signals. The peak of Q-waves is the starting point on the left side of R-waves, and the peak of S-waves is the ending point on the right side of R-waves. The first over-zero point was searched within 20 ms of the left side of the R-wave mode polar pair, which corresponded to the crest of the Q-wave; the first over-zero point was searched within 30 ms of the right side of the R-wave tail mode polar pair, which corresponded to the crest of the S-wave.

**Figure 2:**
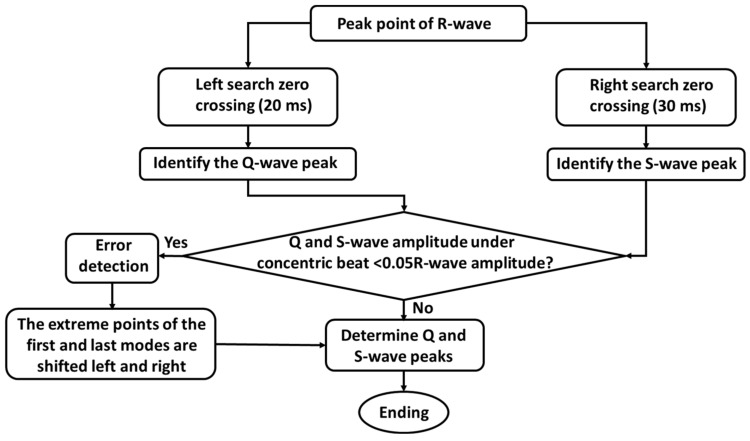
Q-waves and S-waves detection of ECG signals.

False detection occurs when the fetal heart has a lesion that causes the ECG waveform to shift, or when the Q-waves and S-waves are not present. Clinically, it was found that the amplitude of Q-waves and S-waves were generally 0.05 times higher than that of R-waves under concentric beats. If the amplitude was less than 0.05 times, the detection was judged as a false detection. Figure 2 shows the detection of Q-waves and S-waves.

The starting point and ending point of QRS waves correspond to the starting point of Q-waves and ending point of S-waves. The morphologies of starting and ending points are variable, which increases the difficulty of detection. Theoretically, the starting point and ending point of QRS waves correspond to the extreme point of the first mode of Q-waves and the extreme point of the tail mode of S-waves, respectively.

Therefore, a time window of 30 ms and 40 ms was used on the left and right sides of the detected Q-waves and S-waves, respectively, and the left and right mode poles were searched within the time window. The first mode poles searched to the left were the first mode poles corresponding to the Q-waves, and the first mode poles searched to the right were the tail mode poles of the S-waves. The first mode poles of the Q-waves and the last mode poles of the S-waves detected were the starting and ending points of the QRS waves, which were QRS wave duration.

#### 2.3.3. Detection of P-waves, T-waves, and ST Segments

The amplitude of T-waves in the FECG signals is much smaller than that of R-waves in the QRS waves, and their morphologies are diversified. The correct detection of QRS waves can reduce the difficulty of T-wave detection. The T-waves were mainly located on two scales after wavelet transform decomposition, and we calculated its mode polar pair on these two scales.

We detected the mode polar pair generated by the P-waves in the left 1/3 of the R-wave first mode polar pair, and detected the mode polar pair generated by the T-waves in the right 1/2 of the R-wave tail mode polar pair; then we can find the positions of the P-waves and T-waves.

According to the waveform characteristics, the first and last mode polarization points of the T-waves were generated from the starting and ending points of the T-waves, and there was a certain correspondence between the mode polarization pair and the waveform starting point. However, there was actually a certain shift phenomenon. Therefore, the extreme points of the T-waves modes could be moved by three sampling points to the right, which were the starting point of the T-waves. According to the principle of approximate symmetry, we used the obtained starting points and peak points to detect the termination points. In addition, P-waves and T-waves have similar symmetry in morphology, so the starting points and ending points of P-waves could be detected by the same method.

### 2.4. Evaluation Methods

To evaluate the performance of FECG signal extraction, we applied the SNR based on eigenvalues ($SNR_{Eig}$) and the SNR based on cross-relation numbers ($SNR_{RMS}$) [14]:

(4) $$SNR_{Eig\;}=\sqrt{\frac{\gamma_{\max}}{\mathrm{sum}(\gamma )-\gamma_{\max}}},$$

where γ is the *M* eigenvalues of the matrix $U^{T}U$, and $\gamma_{\max}$ is the maximum eigenvalues of the matrix $U^{T}U$; and

(5) $$SNR_{RMS}=\sqrt{\frac{\sigma }{1-\sigma }},$$

where $\sigma =\frac{2}{M(M-1)}\sum {}_{i=0}^{M-2}\sum {}_{k=i+1}^{M-1}f{\left(i\right)}^{T}f(k)$, and f is FECG signals containing QRS waves number [14]. When the SNR is larger, the extracted signal quality is higher and the extraction performance is better.

For evaluating the detection of FECG signals of the ADFECGDB database, fetal head signals were used as a reference standard to calculate sensitivity (*Se*), positive predictive value (*PPV*), and the F1 score:

(6) $$Se=\frac{TP}{TP+FN},$$

(7) $$PPV=\frac{TP}{TP+FP},$$

(8) $$F1=\frac{TP}{TP+FP+FN},$$

where *TP* (true positive) indicates the number of FECG R-peak positions correctly detected, *FP* (false positive) indicates the number of FECG R-peak positions incorrectly detected, and *FN* (false negative) indicates the number of FECG R-peak positions missed [6].

## 3. Results

### 3.1. Extraction of FECG Signals

We compared the FECG signal extraction performance of the proposed method with that of the FastICA algorithm and the SVD algorithm alone. The SNRs using the ADFECGDB database are shown in Table 1. The extraction performance of the proposed algorithm was better than that of the FastICA algorithm and the SVD algorithm alone. The $SNR_{RMS}$ of the best channel signals reached 45.028 dB.

**Figure 3:**
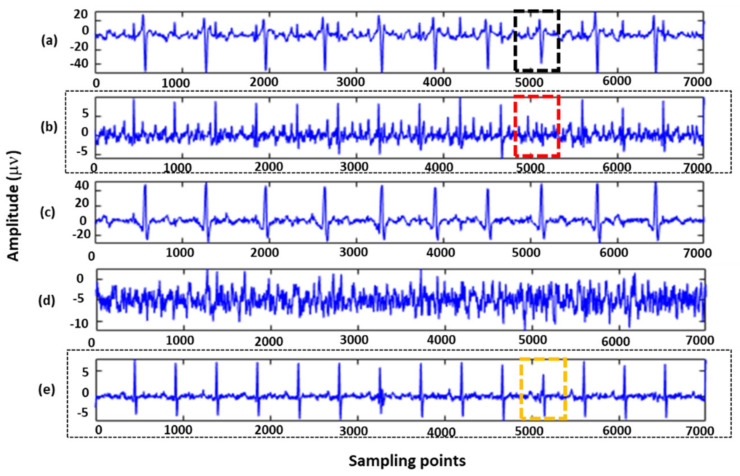
A total of 7000 sampling points of R01 data in the ADFECGDB database were applied to the extraction algorithm: (**a**) The second channel of maternal abdominal mixed signals; (**b**) FECG signals estimated by the SVD algorithm; (**c**) MECG signals extracted by the FastICA algorithm; (**d**) Residual noises assessed by the FastICA algorithm; and FECG signals extracted by the proposed algorithm.

A visualized comparison is shown in Figure 3. The signal quality of Figure 3e was significantly better than that of Figure 3b, as the noise in Figure 3e was significantly improved. In addition, when maternal–fetal signals overlapped (indicated by the black box), a single algorithm led to FECG signal missing waveforms (indicated by the red box), which were addressed by using the proposed method (indicated by the yellow box).

**Figure 4:**
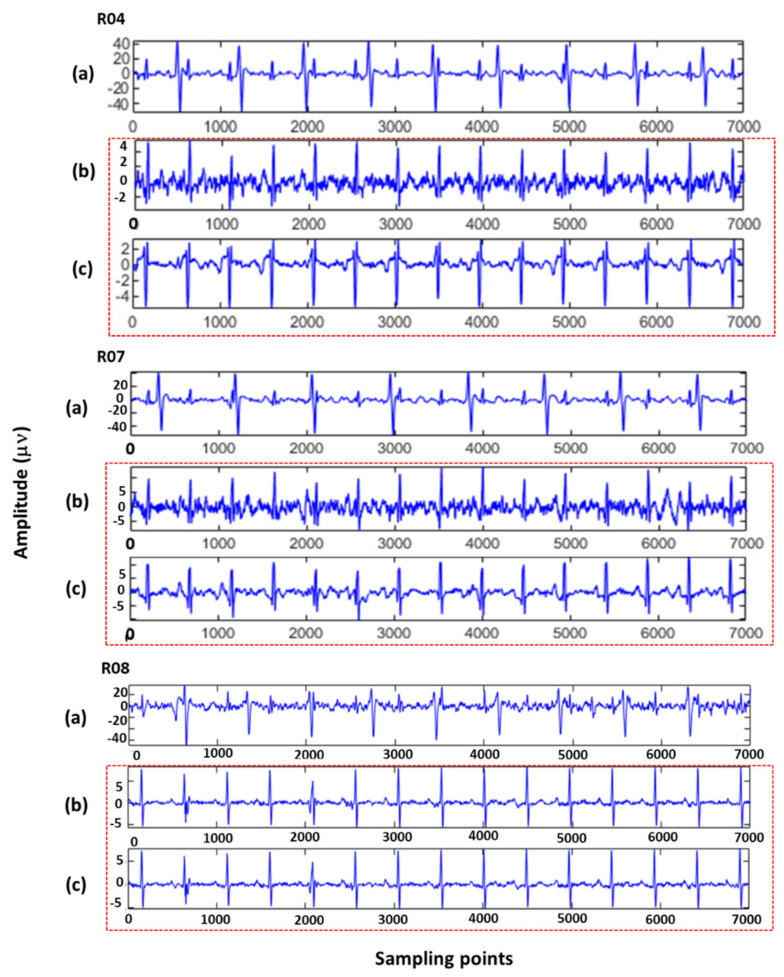
A total of 7000 sampling points of R04, R07, and R08 data in the ADFECGDB database were applied to the extraction algorithm: (**a**) Maternal abdominal mixed signals (R04, R07, and R08 signals were, respectively, used as the maternal abdominal mixed signals of the third, third and second channels); (**b**) FECG signals estimated by the SVD algorithm; and (**c**) FECG signals extracted by the proposed algorithm.

Figure 4 compares FECG signal extraction between the proposed method and the SVD algorithm. The quality of the FECG signals extracted by the proposed method was obviously better than that extracted by the SVD algorithm.

**Figure 5:**
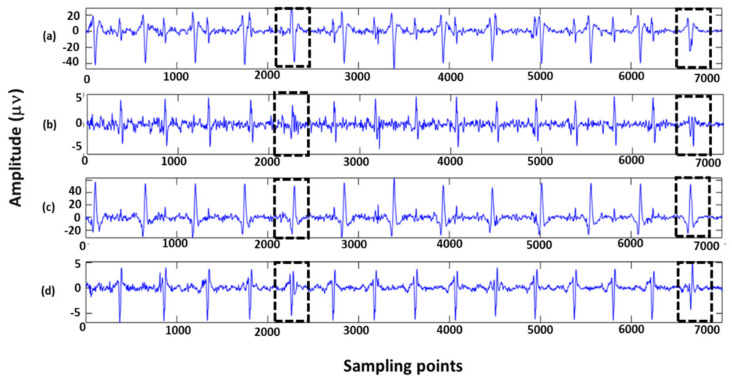
A total of 7000 sampling points of R10 data in the ADFECGDB database were applied to the extraction algorithm: (**a**) The second channel of maternal abdominal mixed signals in R10 data; (**b**) FECG signals estimated by the SVD algorithm; (**c**) MECG signals extracted by the FastICA algorithm; and (**d**) FECG signals extracted by the proposed algorithm.

Figure 5 shows that the quality of the extracted FECG signals are significantly enhanced by the proposed method, and the issue of missing waveforms is solved when maternal–fetal signals overlap (indicated by the black box).

In order to further validate the proposed algorithm, we used 10 datasets from the PhysioNet2013 database. The SNRs are shown in Table 2. The SNRs of the signals extracted by the proposed algorithm were higher.

### 3.2. Detection of FECG Signals

For the detection of FECG signals, we used the ADFECGDB database to verify the detection algorithm. The results of correct detection, missed detection, and incorrect detection of R-waves are shown in Table 3. The average value of *Se*, *PPV*, and F1 were 96.90%, 98.23%, and 95.24%, respectively.

**Figure 6:**
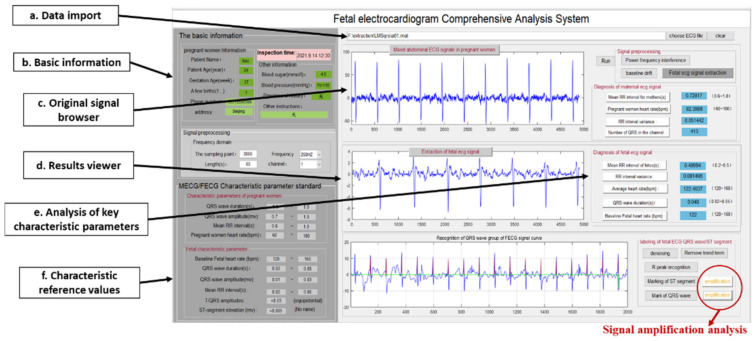
The FECG signal analysis GUI mainly consisted of six parts: (a) Data import; (b) Basic information; (c) Original signal browser; (d) Results viewer; (e) Analysis of key characteristic parameters; and (f) Characteristic reference values.

In addition, we used the MATLAB GUI development environment to develop a user-friendly GUI (Figure 6) for FECG signal extraction, including data import, signal preprocessing, FECG signal extraction, and key characteristic parameter detection and marking. Signal preprocessing included removing noises such as baseline drift, power frequency interference, and pulse trace from the initial mixed ECG signals. Calculation of characteristic parameters included real-time calculation of maternal heart rates, R-R intervals, fetal R-R intervals, QRS durations, fetal heart rates, and other parameters.

**Figure 7:**
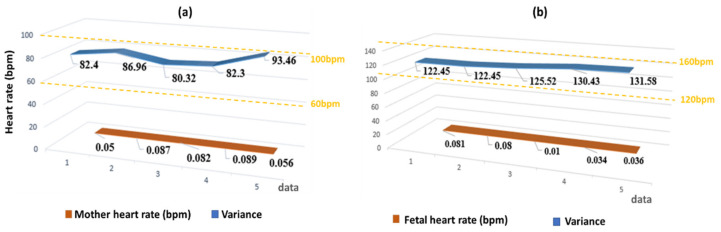
Evaluation of the accuracy of maternal and fetal heart rate calculations using variances. The blue curves in (**a**) and (**b**) were maternal and fetal heart rates calculated, respectively. The orange curves in (**a**) and (**b**) represented the difference between the heart rate and the real heart rate of each channel. The yellow dotted lines represented, respectively, the reference range of normal heart rates of the mother and the fetus.

The R-R intervals, heart rates, and other values calculated from five datasets in the ADFECGDB database are shown in Table 4. The parameters calculated were all within the normal range, and the variance was less than 0.1 when compared with the real heart rate. A more intuitive line chart is shown in Figure 7.

**Figure 8:**
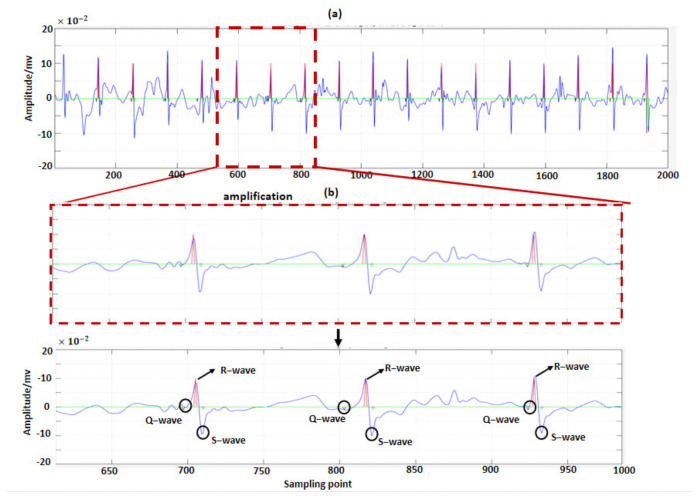
Detection of QRS waves (using R01 data from the ADFECGDB database): (**a**) is the detection results of QRS waves; (**b**) is the amplification marker of (**a**).

We used the wavelet mode maximum method to detect QRS waves and ST segments of FECG signals. The results of QRS wave detection are shown in Figure 8.

**Figure 9:**
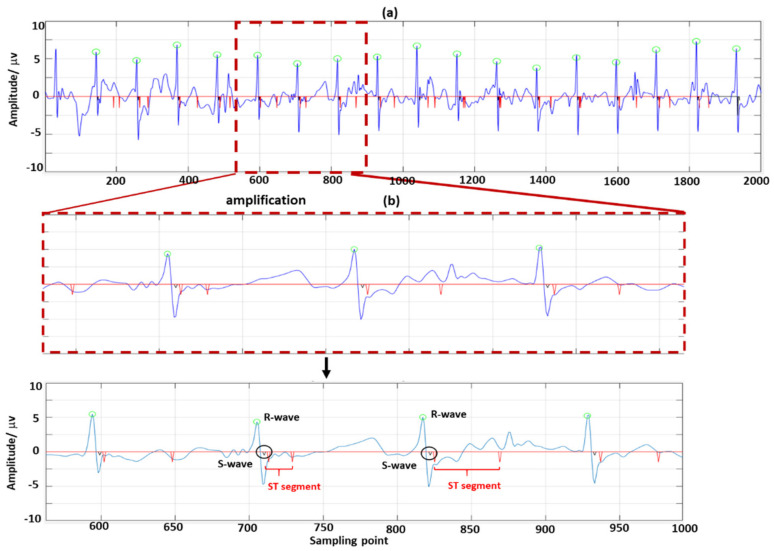
Detection of ST segments (using R01 data from the ADFECGDB database): (**a**) is the detection results of ST segments; (**b**) is the amplification marker of (**a**).

Changes in the ST segments are an important indicator to assess whether the fetus is healthy [26]. The results of T-wave detection are shown in Figure 9. The T-wave morphology was diverse and the amplitudes were smaller compared to that of the QRS waves. There was variability in the detection and identification of the ST segments.

## 4. Discussion

### 4.1. Significance of This Study

In this work, we proposed a method for the extraction of FECG signals by combining FastICA and SVD algorithms. Singular values in SVD correspond to important information implied in the matrix, and the sum of the top 1% singular values may account for more than 99% of the total singular values. We chose the two largest singular values and the corresponding left and right singular vectors to approximate the characteristic matrix of FECG signals. Then, the SVD algorithm was combined with the FastICA algorithm to extract FECG signals. Using the ADFECGDB and PhysioNet2013 databases for validation, the SNR of the best channel signal reached 45.028 dB and the R-peak amplitude was almost unattenuated. In addition, the issue of missing waveforms was addressed.

We employed the wavelet mode maxima method to detect QRS waves and ST segments of FECG signals. We used the orthogonal quadratic spline wavelet to decompose FECG signals into four scales to obtain detailed information. The energy of R-waves, Q-waves, and S-waves was the strongest at the fourth scale, and that of T-waves was the strongest at the second scale. Therefore, we searched the maximum pair of modes at the fourth scale and the second scale. Then, the zeroes and peak point positions were obtained. we compared the detected R-peak positions with the reference positions, and the *Se, PPV*, and F1 score were 96.90%, 98.23%, and 95.24%, respectively. Finally, we designed a GUI that integrated the proposed extraction and detection algorithms to provide systematic support for fetal health evaluation.

### 4.2. Comparison with Related Work

Compared with previous work [14], under the same number of iterations, the SNRs based on eigenvalues and cross-relation numbers have been improved. Table 5 shows the performance comparison between the proposed algorithm and state-of-the-art methods. The proposed algorithm is similar to Refs. [12,28]. Sarafan et al. [12] proposed a method combining ICA, TS, and EKF. They used three different ICA algorithms to obtain MECG and FECG estimates, and then used EKF to filter out residual MECG components, with an F1 score of 92.61%. Similarly, Panigrahy and Sahu [28] used EKS to estimate MECG signals. As the relationship between FECG signals and MECG components in the mixed signals was nonlinear, they used an adaptive neuro fuzzy inference system (ANFIS) to estimate the actual MECG component. Experimental results showed that our proposed algorithm combining FastICA and SVD had a higher accuracy. The FastICA algorithm uses negative entropy to measure the non-Gaussian properties of the signal, which effectively combines the algorithm characteristics of fixed point iteration and the statistical characteristics of negative entropy, making the algorithm simple and fast for convergence.

In recent years, many researchers have applied deep learning algorithms to ECG signals processing [29,30]. In the analysis of FECG signals, signal features were identified by constructing neural networks in Refs. [3,16,17,31]. As shown in Table 5, when detecting R-waves, the performance of these algorithms were similar to that of the proposed algorithm. However, for the extraction of low-amplitude signal features, such as P, S, and T waves, deep learning algorithms may be more efficient and perform better in identifying detailed features. In addition, deep learning algorithms do not need to manually select specific signal features, and can automatically learn appropriate features. In Ref. [3], Fotiadou et al. used convolutional neural network blocks as feature extractors to automatically extract signal features and directly determine fetal heart rate. Compared with the proposed algorithm, the disadvantage of these network models may lie in the fact that the generalization ability could not be increased simply by adding more training samples. On the other hand, when the training data contain samples from different databases, the accuracy of the deep learning methods may be improved.

**Table 5:** Comparison with published state-of-the-art methods for FECG signals extraction.

Authors	Methods	Year	Database	*Se/%* *	*PPV/%*	F1*/%*
Gurve et al. [32]	ICA	2020	ADFECGD and PhysioNet2013	93.30	94.00	93.60
Gurve et al. [32]	NNMF + ICA	2020	ADFECGD and PhysioNet2013	95.30	94.60	94.80
Taha et al. [5]	FastICA	2020	PhysioNet2013	97.30	93.30	95.70
Barnova et al. [6]	EEMD	2021	ADFECGD and PhysioNet2013	81.79	87.16	84.08
Barnova et al.[6]	EEMD + RLS + ICA	2021	ADFECGD and PhysioNet2013	95.09	96.36	95.69
Sarafan et al. [12]	FastICA + TS + EKF	2020	PhysioNet2013	-	-	92.61
Zhang et al.[33]	K-means + PCA	2019	PhysioNet2013	96.23	95.35	95.78
Panigrahy et al.[28]	EKS + DE + ANFIS	2017	PhysioNet2013	91.47	92.18	-
Jaba et al. [34]	PSF + ANC	2021	DaISy and PhysioNet2013	97.92	94.66	96.12
Liu et al. [35]	SQA + FTM	2014	PhysioNet2013	94.13	93.74	93.90
Mollakazemi et al. [36]	PCA + WT	2021	PhysioNet2013	-	-	98.77
Jallouli et al. [27]	Clifford Wavelet Entropy	2021	PhysioNet2013	99.76	99.2	99.47
Rasti-Meymandi et al. [37]	AECG-DecompNet	2021	PhysioNet2013	97.40	93.52	95.42
Fotiadou et al. [3]	DNN+ LSTM	2020	PhysioNet2013	98.10	-	-
Vo et al. [16]	1-D Octave convolution	2020	PhysioNet2013	-	-	90.70
Ting et al. [17]	2-D-CNN	2021	PhysioNet2013	95.20	-	-
Mohebian et al. [31]	Conv1D + CycleGAN	2021	ADFECGDand NI-FECG	-	-	99.70
Our method	FastICA + SVD + WT	2022	ADFECGD and PhysioNet2013	96.90	98.23	95.24

* *Se* is sensitivity, *PPV* is positive predictive value, and F1 score is the harmonic average of accuracy and recall.

### 4.3. Limitations and Future Work

This study has limitations. Firstly, we only used public data to verify the accuracy of the algorithm. In future work, we will use the abdominal electrode method to collect real data to verify the algorithm. Secondly, deep learning methods can extract deep features and identify the details of signals effectively. In future work, we can apply these algorithms to analyze the FECG signals. For instance, a lightweight network model can be designed to extract FECG signals efficiently. Lastly, infant physical conditions and signal sampling locations may affect the intensity and characteristics of FECG signals and noises. Multi-channel MECG signals at different sampling locations contain different FECG information. Therefore, we plan to integrate multi-dimensional signals into the system and analyze multi-channel signals simultaneously. As such, the fetus health can be evaluated more comprehensively.

## 5. Conclusions

FECG has become an important means of recognizing fetal heart activity and fetal diseases. However, the FECG signal is extremely weak and easily interfered by noises, so the extraction of clean FECG signals is important and challenging in clinical practice. In this paper, we proposed an improved FastICA and SVD algorithm to extract FECG signals and a wavelet mode maximum method to detect QRS waves and ST segments of FECG signals. These algorithms were integrated into a GUI. The proposed method may be used as a new method for FECG signals extraction and detection. In future work, deep learning methods and multi-channel signals may be considered.

## Figures and Tables

**Table 1:** Performance comparison of FECG signal extraction algorithms using the ADFECGDB database.

Data	FastICA		SVD		FastICA + SVD	
	$SNR_{Eig}$	$SNR_{RMS}$	$SNR_{Eig}$	$SNR_{RMS}$	$SNR_{Eig}$	$SNR_{RMS}$
R01	1.158	0.369	2.982	6.956	3.194	7.759
R04	1.549	0.184	2.494	5.265	2.749	6.637
R07	2.305	5.125	1.872	3.391	5.349	26.632
R08	1.072	0.142	1.186	9.682	3.125	45.028
R10	2.927	7.797	2.873	5.118	3.258	10.362

**Table 2:** Performance comparison of FECG signal extraction algorithms using the PhysioNet2013 database.

Data	FastICA		SVD		FastICA + SVD	
	$SNR_{Eig}$	$SNR_{RMS}$	$SNR_{Eig}$	$SNR_{RMS}$	$SNR_{Eig}$	$SNR_{RMS}$
a01	0.739	0.051	0.732	0.342	2.296	4.073
a03	1.281	1.394	1.787	2.177	1.796	2.183
a04	0.992	0.112	1.122	1.081	2.018	3.185
a06	0.538	−0.644	0.747	0.220	3.197	7.529
a08	1.071	0.142	1.185	0.681	3.120	7.767
a10	0.793	0.093	1.261	0.557	4.357	11.178
a12	1.067	0.900	1.492	1.138	14.556	11.202
a13	7.146	1.007	5.773	0.668	9.461	5.916
a17	6.324	1.188	6.242	0.982	9.159	2.734
a21	1.058	0.749	0.648	0.088	4.046	15.640

**Table 3:** FECG signal detection performance evaluated by five datasets from the ADFECGDB database.

Data	Correct Detection	Missing Detection	Incorrect Detection	*Se/%* *	*PPV/%*	F1*/%*
R01	627	17	2	97.36	99.68	97.06
R04	620	12	19	98.10	97.02	95.24
R07	613	14	21	97.77	96.69	94.60
R08	623	19	5	97.04	99.20	96.29
R10	620	37	9	95.65	98.57	93.09
average	3103	99	56	96.90	98.23	95.24

* *Se* is sensitivity, *PPV* is positive predictive value, and F1 score is the harmonic average of accuracy and recall.

**Table 4:** Calculation of key characteristic parameters.

Data	Maternal Characteristic Parameters			Fetal Characteristic Parameters			
	MECG QRS Numbers	Mean R-R Intervals (s)	Heart Rates (bpm)	FECG QRS Numbers	Mean R-R Intervals (s)	Fetal Heart Rates (bpm)	Baseline Fetal Heart Rates (bpm)
R01	413	0.728	82.40	644	0.490	122.45	122
R04	433	0.690	86.96	632	0.490	122.45	122
R07	401	0.747	80.32	627	0.478	125.52	126
R08	411	0.729	82.30	642	0.460	130.43	130
R10	480	0.642	93.46	657	0.456	131.58	132

## Data Availability

The data used to support the findings of this study are included in the article.
